# Impact of body mass index on in-hospital mortality for six acute cardiovascular diseases in Japan

**DOI:** 10.1038/s41598-022-23354-y

**Published:** 2022-11-07

**Authors:** Naofumi Yoshida, Masato Ogawa, Michikazu Nakai, Koshiro Kanaoka, Yoko Sumita, Takuo Emoto, Yoshihiro Saito, Hiroyuki Yamamoto, Kazuhiro P. Izawa, Yoshitada Sakai, Yushi Hirota, Wataru Ogawa, Yoshitaka Iwanaga, Yoshihiro Miyamoto, Tomoya Yamashita, Ken-ichi Hirata

**Affiliations:** 1 Division of Cardiovascular Medicine, Department of Internal Medicine, Kobe University Graduate School of Medicine, 7-5-1 Kusunoki-cho, Chuo-ku, Kobe, 6500017 Japan; 2 Division of Rehabilitation Medicine, Kobe University Hospital, Kobe, 6500017 Japan; 3 Department of Public Health, Graduate School of Health Sciences, Kobe University, Kobe, 6540142 Japan; 4 Department of Medical and Health Information Management, National Cerebral and Cardiovascular Center, Osaka, 5648565 Japan; 5 Division of Diabetes and Endocrinology, Department of Internal Medicine, Kobe University Graduate School of Medicine, Kobe, 6500017 Japan

**Keywords:** Cardiology, Medical research, Risk factors

## Abstract

Body mass index (BMI) distribution and its impact on cardiovascular disease (CVD) vary between Asian and western populations. The study aimed to reveal time-related trends in the prevalence of obesity and underweight and safe ranges of BMI in Japanese patients with CVD. We analyzed 5,020,464 records from the national Japanese Registry of All Cardiac and Vascular Diseases—Diagnosis Procedure Combination dataset over time (2012–2019) and evaluated BMI trends and the impact on in-hospital mortality for six acute CVDs: acute heart failure (AHF), acute myocardial infarction (AMI), acute aortic dissection (AAD), ischemic stroke (IS), intracerebral hemorrhage (ICH), and subarachnoid hemorrhage (SAH). Patients were categorized into five groups using the WHO Asian-BMI criteria: underweight (< 18.5 kg/m^2^), normal (18.5–22.9 kg/m^2^), overweight at risk (23.0–24.9 kg/m^2^), obese I (25.0–29.9 kg/m^2^), and obese II (≥ 30.0 kg/m^2^). Age was significantly and inversely related to high BMI for all diseases (P < 0.001). The proportion of BMI categories significantly altered over time; annual BMI trends showed a significant and gradual increase, except AAD. In adjusted mixed models, underweight was significantly associated with a high risk of in-hospital mortality in all CVD patients (AHF, OR 1.41, 95% CI 1.35–1.48, P < 0.001; AMI, OR 1.27, 95% CI 1.20–1.35, P < 0.001; AAD, OR 1.23, 95% CI 1.16–1.32, P < 0.001; IS, OR 1.45, 95% CI 1.41–1.50, P < 0.001; ICH, OR 1.18, 95% CI 1.13–1.22, P < 0.001; SAH, OR 1.17, 95% CI 1.10–1.26, P < 0.001). Moreover, obese I and II groups were significantly associated with a higher incidence of in-hospital mortality, except AHF and IS. Age was associated with in-hospital mortality for all BMI categories in six CVD patients. BMI increased annually in patients with six types of CVDs. Although underweight BMI was associated with high mortality rates, the impact of obesity on in-hospital mortality differs among CVD types.

## Introduction

Obesity is associated with an increased risk of developing individual cardiovascular diseases (CVDs) such as heart failure and arteriosclerotic disease and their risk factors such as hypertension, dyslipidemia, and type 2 diabetes^1,2^. Obesity is additionally associated with the risk of cardiometabolic multimorbidity—2–10 times higher than that for healthy body mass index (BMI)—and increased risk of mortality^3–5^. As the prevalence of obesity has increased worldwide in the past two decades^4,6^, obesity-related CVD and associated medical costs have risen dramatically and are expected to rise further^7^. Considering this evidence that obesity must be a grave public health threat, it is natural that many countries including America, Europe, and Asian countries have been paying attention to the global pandemic of obesity.

Contradictory evidence shows that obesity is protective and associated with greater survival in certain groups of patients with CVD, particularly heart failure—the “obesity paradox”^8^. This indicates that uncertainties regarding the causal status and prognostic markers of BMI for CVD remain to be eliminated. The optimal cut-off for obesity using BMI in Asian population could be different from that in the western population^9^. Research on Asian populations using Asian obesity criteria is crucial to yield better understanding of the specific Asian-related disease phenotypes. However, trends in BMI distribution and the burden of obesity on mortality risk in acute CVD remain uncertain.

Being underweight is an independent predictor of increased morbidity and mortality in patients with CVD^10^ because it is considered a consequence of body wasting (i.e., cardiac cachexia). Underweight status is especially common in the elderly^11^ and is garnering attention in the aging society of Japan. Thus, understanding the extent to which underweight patients account for each CVD over time and its impact on in-hospital mortality is important to clarify the current situation and to predict future implications of cardiovascular medicine.

The present study aimed to establish evidence for BMI distribution and determine the impact of BMI on mortality in CVDs in the aging society of Japan. To achieve this goal, we analyzed a Japanese nationwide administrative database of patients with six major acute CVDs, namely, acute heart failure (AHF), acute myocardial infarction (AMI), acute aortic dissection (AAD), ischemic stroke (IS), intracerebral hemorrhage (ICH), and subarachnoid hemorrhage (SAH) over time and evaluated BMI trends from underweight to obesity and the impact on all-cause in-hospital mortality. These results might help provide risk stratification using BMI, provide promising intervention strategies stratified by BMI, and establish medical policies focused on BMI to create a sustainable society. These objective data also help healthcare providers in daily clinic to manage patients. Furthermore, as the Asian population is aging rapidly^12^, data from Japan, a super-aged society, may be clinically relevant and informative for other Asian countries.

## Methods

### Data source

The Japanese Registry Of All Cardiac and Vascular Diseases and the Diagnosis Procedure Combination (JROAD-DPC) is a nationwide registry established by the Japanese Circulation Society (JCS). Details of the JROAD-DPC dataset have been described elsewhere^13–16^. Briefly, the JROAD database covers nearly all teaching hospitals with cardiovascular beds because this survey is mandatory at JCS-certified teaching hospitals. Among them, more than 1000 hospitals provided DPC data to the JROAD-DPC. It contains a medical database on patient information such as age, sex, height, weight, diagnosis, comorbidities at admission, admission and discharge date, discharge outcome, clinical examinations and treatment status, and hospital overview but does not contain the cause of death or test data of laboratory and physiological functions. The main diagnoses and comorbidities are coded using the International Classification of Disease and Related Health Problems 10th Revision (ICD-10) codes.

Patient information in the JROAD-DPC dataset can only be linked when the patient is admitted to the same hospital because different identifiers are assigned to patients by different hospitals within the database. Because of the anonymous nature of the data, the requirement for informed consent was waived by the Ethics Committee of Kobe University. The study was conducted in accordance with the principles of the Declaration of Helsinki and approved by the Ethics Committee of Kobe University (no. B210052).

### Study population

The JROAD-DPC database included 5,020,464 health records from 1086 JCS-certified teaching hospitals between April 2012 and March 2020. A detailed strategy for selecting the records to be analyzed is shown in Supplementary Fig. 1. We identified records for each CVD from the JROA-DPC database when relevant ICD-10 codes appeared either in the main diagnosis, admission precipitating diagnosis, or most resource-consuming diagnosis since these records linked to diagnosis. The following ICD-10 codes were used to extract each disease: AMI (I21), AHF (I50), AAD (I71.0), IS (I63), ICH (I61), and SAH (I60). AHF was further defined using an additional code 30101 or 30102. Comorbidities were determined primarily from the following ICD-10 codes: hypertension (HT) (I10-I13, I15), diabetes mellitus (DM) (E10-E14), dyslipidemia (DL) (E78), atrial fibrillation/flutter (AF) (I48), chronic kidney disease (CKD) (N18), and chronic respiratory disease (CRD) (I27, J40-J45, J47, J60-J67, J70). Comorbidities were also checked against the medications and procedures each patient received or underwent to determine consistency with the code data.

Only patients admitted to the emergency department and those hospitalized for treatment were included. We excluded readmission records to investigate the impact of BMI on in-hospital mortality based on patients and *not* on their multiple admission records; we also excluded patients aged < 20 years. We further excluded patients whose length of hospital stay was ≤ 1 day because their medical histories might not have been properly recorded. Patients without Killip classification data for AMI and New York Heart Association (NYHA) data for HF were also excluded to ensure data quality^17,18^. Finally, we excluded hospitals with ≤ 10 cases for each CVD diagnosis, consistent with a previous study by the Center for Medicare and Medicaid Service that maintained anonymous data and excluded small case volumes^19^. For three cerebrovascular diseases (IS, ICH, and SAH), data were included from April 2015 when the register was started on a national basis.

The JROAD-DPC data were collected in accordance with the Japanese fiscal year; therefore, we used the Japanese fiscal year in the analysis. For instance, data of “year 2012” consisted of data from April 2012 to March 2013.

### BMI and categorization

BMI is defined as body weight divided by the square of the body height, expressed in units of kg/m^2^. We categorized patients according to the World Health Organization (WHO) Asian-BMI classification^20^ as follows: underweight, BMI < 18.5; normal range, 18.5 ≤ BMI < 23; overweight at risk, 23 ≤ BMI < 25; obese category I, 25 ≤ BMI < 30; and obese category II, 30 ≤ BMI. For the purpose of this study, overweight at risk, obese I, and obese II were all considered obese.

### Statistical analysis

Statistical analyses were performed using Stata 16.1 (Stata Corp, College Station, TX, USA). The Shapiro–Wilk test was used to determine whether the data were normally distributed. Results were expressed as mean ± standard error of the mean for normally distributed data. The χ^2^ test was used to compare categorical variables. One-way analysis of variance was used to compare the means of more than two groups. Patients without records of height or weight and those with abnormal BMI (10.0 kg/m^2^ < BMI or BMI > 60 kg/m^2^) were considered “missing”. Multiple imputation methods by the chained equation algorithm using two variables (BMI and age) were applied based on 10 replications, assuming missing at random mechanisms. The Cochran-Armitage test was used to test whether there was a linear BMI trend over time. Multilevel mixed-effect logistic regression analysis using institution as a random intercept was performed to examine the association between the incidence of in-hospital mortality and each variable. To calculate odds ratios (ORs) and 95% confidence intervals (CIs) for in-hospital mortality, age and sex were simultaneously entered into a multivariable logistic regression model (model 1). Additionally, factors theoretically related to in-hospital mortality, such as HT, DM, DL, CKD, CRD, and AF, as well as age and sex, were simultaneously entered into a multivariable logistic regression model (model 2). OR and 95% CI for in-hospital mortality were calculated for each BMI category, with respect to the reference value of normal BMI. The OR, 95% CI, and P-values for the interaction were also calculated among three age subgroups (20 ≤ age < 65, 65 ≤ age < 75, and 75 ≤ age) in model 2, with respect to the reference value of 20 ≤ age < 65 with normal BMI. Statistical significance was set at P < 0.05.

## Results

### Study population and baseline patient characteristics

**Figure 1:**
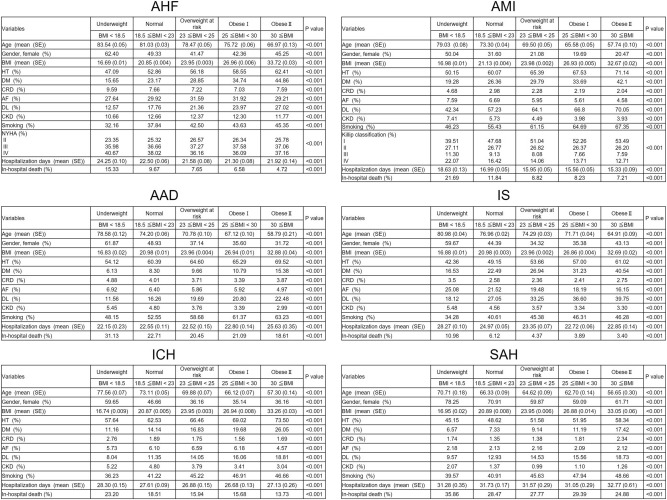
Patients’ characteristics for each indicated cardiovascular disease (CVD). Patients’ clinical background, hospitalization days, and rates of all-cause in-hospital mortality for the indicated cardiovascular diseases are shown. *AAD* acute aortic dissection, *AF* atrial fibrillation/flutter, *AHF* acute heart failure, *AMI* acute myocardial infarction, *BMI* body mass index, *CKD* chronic kidney disease, *CRD* chronic respiratory disease, *DL* dyslipidemia, *DM* diabetes mellitus, *HT* hypertension, *ICH* intracerebral hemorrhage, *IS* ischemic stroke, *SAH* subarachnoid hemorrhage.

The final sample size included in the analysis was as follows: AHF, n = 277,489; AMI, n = 307,295; AAD, n = 96,114; IS, n = 588,382; ICH, n = 201,243; and SAH, n = 62,420 (Supplementary Fig. 1). Patients were divided into five groups according to the WHO Asian-BMI classification (Fig. 1). In all diseases, age was significantly and inversely related to the high BMI groups (P < 0.001). Surprisingly, the mean age of underweight patients was 83.54 years for AHF, reflecting the current status of Japan’s super-aged society. Females were more common in the underweight group for all CVDs; in the underweight group, more than half were female (AHF, 62.40%; AMI, 50.04%; AAD, 61.87%; IS, 59.67%; ICH, 59.65%; and SAH, 78.25%). Contrarily, more than half of the obese patients (overweight at risk, obese I, and obese II) with AHF, AMI, AAD, IS, and ICH were males. This trend was most prominent in AMI (percentage of men: overweight at risk, 88.92%; obese I, 80.31%; obese II, 79.53%). The proportions of patients with HT, DM, CRD, AF, DL, CKD, and smoking in each BMI group were significantly different among the five BMI groups for all diseases (P < 0.001). Lifestyle-related diseases such as HT, DM, and DL were more common in obese patients (overweight at risk, obese I, and obese II) than in the underweight group for all diseases. Distribution of NYHA and Killip classification in each BMI group was also significantly different among the five BMI groups. Hospitalization days varied widely among each disease; patients with AMI needed the shortest hospitalization, whereas those with SAH needed the longest hospitalization. Patients in the underweight group had longer hospitalization days for AHF, AMI, IS, and ICH.

### In-hospital mortality

The rates of all-cause in-hospital mortality for each disease are shown in Fig. 1. For all BMI groups, highest in-hospital mortality occurred in SAH (underweight, 35.86%; normal, 28.47%; overweight at risk, 27.77%; obese I, 29.39%; obese II, 24.88%), whereas the lowest occurred in IS (underweight, 10.98%; normal, 6.12%; overweight at risk, 4.37%; obese I, 3.89%; obese II, 3.40%). In all diseases, the underweight group exhibited the highest in-hospital mortality. Specifically, in AHF, AMI, IS, and ICH, the high BMI groups showed less in-hospital mortality.

### Annual trends of BMI

**Figure 2:**
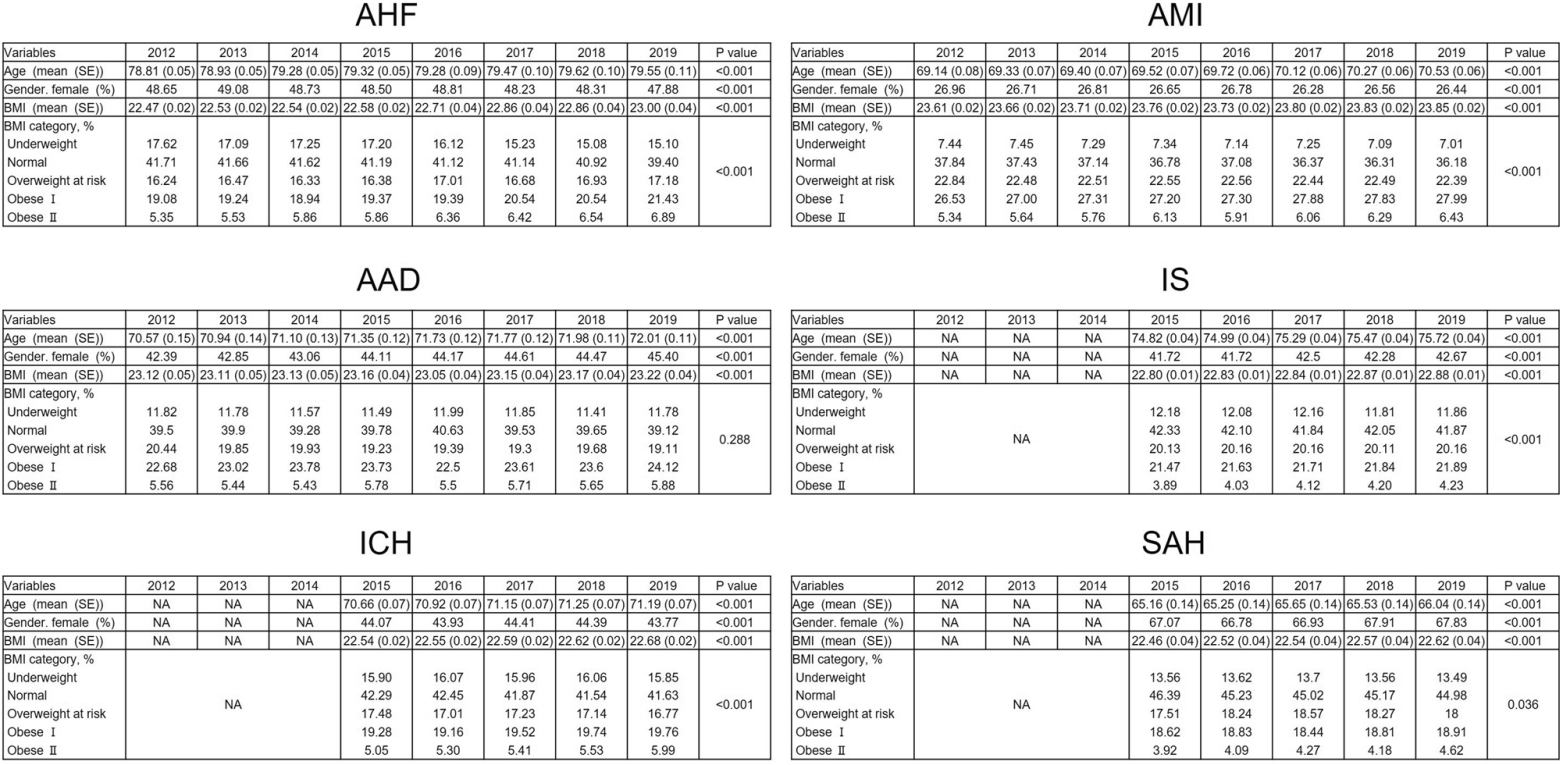
Trends in BMI categories and ages over time in the indicated cardiovascular disease (CVD) groups. Trends in BMI categories and ages from 2012 to 2019 in AHF, AMI, AAD and from 2015 to 2019 in IS, ICH, and SAH are shown. The Cochran-Armitage test was used to assess BMI trends over time. *AAD* acute aortic dissection, *AHF* acute heart failure, *AMI* acute myocardial infarction, *BMI* body mass index, *ICH* intracerebral hemorrhage, *IS* ischemic stroke, *NA* not applicable, *SAH* subarachnoid hemorrhage.

Figure 2 shows the annual trends of age, sex, mean BMI, and BMI categories in the six CVD groups. In all diseases, there were significant age differences, and the mean age of patients tended to increase slightly over time. Among the six diseases, patients with SAH were the youngest, while those with AHF were the oldest. There were significant differences in sex over time; the proportion of female patients increased AAD and IS but not in other diseases.

Regarding to the BMI trend over time, although the mean BMI significantly changed over time in all diseases, it fell within the range of 22–24 and did not exceed 25. The normal BMI group was the major component in all diseases every year, accounting for approximately 40–45%. Obese I was the second major BMI group in all diseases every year, except SAH 2017. Importantly, the percentage of obese II patients gradually increased annually in AHF, IS, and ICH and tended to increase in AMI, AAD, and SAH. Similarly, the proportion of obese I patients tended to increase in AHF, AMI, and IS. Conversely, the percentage of underweight patients drastically decreased in AHF and tended to decrease slightly in AMI and IS. No obvious trend was observed in the proportion of underweight patients in the rest of the CVDs. Regarding the overweight at risk group, a unique BMI group in the WHO Asian classification, a consistent downward or upward trend was not observed in our study period; an increasing tendency was not observed in any disease. Putting these together, statistical analysis showed significant percentage trends of each BMI category over time in AHF, AMI, IS, ICH, and SAH (AHF, P < 0.01; AMI, P < 0.01; AAD, P = 0.288; IS, P < 0.01; ICH, P < 0.01, SAH, P = 0.036). We also observed a significant decrease in percentage of underweight patients over time in AHF, AMI, and IS, whereas no significant trend was observed in AAD, ICH, and SAH (AHF, P < 0.01; AMI, P < 0.01; AAD, P = 0.95, IS, P < 0.01; ICH, P = 0.858; SAH, P = 0.777).

### Impact of BMI on in-hospital mortality

**Figure 3:**
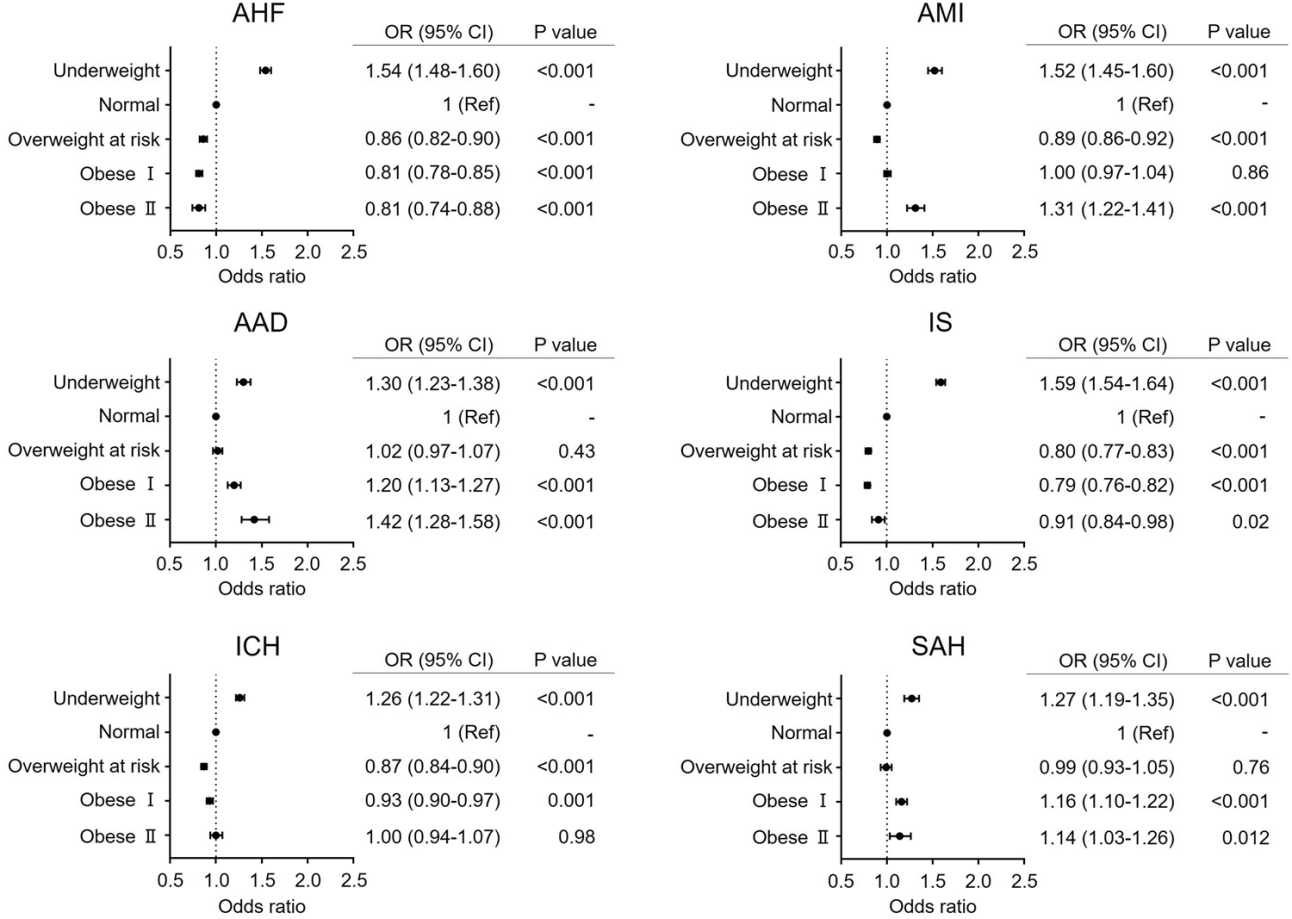
Impact of BMI on in-hospital mortality in each cardiovascular disease (CVD) group: model 1. Age and sex were simultaneously entered into a multivariable logistic regression model to investigate the impact of BMI on in-hospital mortality. Only odds ratios (ORs) and 95% confidence intervals (CIs) of each BMI category are shown. *AAD* acute aortic dissection, *AHF* acute heart failure, *AMI* acute myocardial infarction, *BMI* body mass index, *CI* confidence interval, *ICH* intracerebral hemorrhage, *IS* ischemic stroke, *OR* odds ratio, *SAH* subarachnoid hemorrhage.

To investigate the impact of BMI on mortality during hospitalization, multilevel mixed-effect logistic regression analyses were performed. After adjusting for age and sex (model 1), we found that the impact of BMI on the risk of hospital death varied among patients with CVD (Fig. 3). Notably, the underweight group was consistently associated with a significantly higher risk of in-hospital mortality in all CVDs than the normal BMI group (AHF, OR 1.54, 95% CI 1.48–1.60, P < 0.001; AMI, OR 1.52, 95% CI 1.45–1.60, P < 0.001; AAD, OR 1.30, 95% CI 1.23–1.38, P < 0.001; IS, OR 1.59, 95% CI 1.54–1.64, P < 0.001; ICH, OR 1.26, 95% CI 1.22–1.31, P < 0.001; SAH, OR 1.27, 95% CI 1.19–1.35, P < 0.001). The overweight at risk group did not show significantly increased risk for in-hospital mortality in six CVDs but rather showed significantly lower risk for in-hospital mortality than the normal BMI group with AHF, AMI, IS, and ICH (AHF, OR 0.86, 95% CI 0.82–0.90, P < 0.001; AMI, OR 0.89, 95% CI 0.86–0.92, P < 0.001; IS, OR 0.80, 95% CI 0.77–0.83, P < 0.001; ICH, OR 0.87, 95% CI 0.84–0.90, P < 0.001). In AMI, AAD, and SAH, the obese II group showed a significantly higher risk for in-hospital mortality than the normal group (AMI, OR 1.31, 95% CI 1.22–1.41, P < 0.001; AAD, OR 1.42, 95% CI 1.20–1.58, P < 0.001; SAH, OR 1.14, 95% CI 1.03–1.26, P = 0.012). Contrarily, in AHF, the obese II group showed a significantly lower risk of in-hospital mortality than the normal group (OR 0.81, 95% CI 0.74–0.88, P < 0.001), which is known to be the obesity paradox. This paradox was also observed in the IS group (OR 0.91, 95% CI 0.84–0.98, P = 0.02).

**Figure 4:**
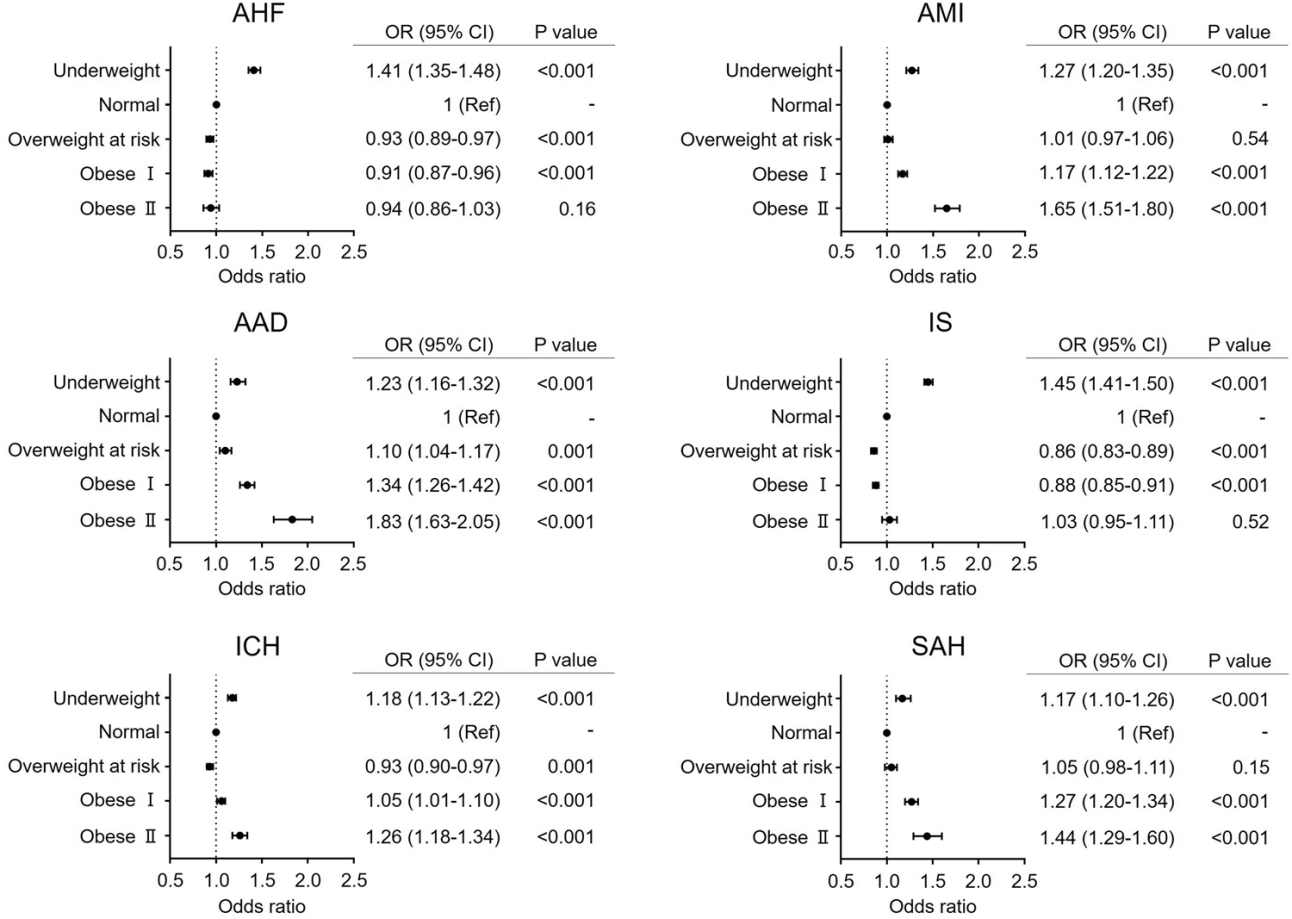
Impact of BMI on in-hospital mortality in each cardiovascular disease (CVD) group: model 2. HT, DM, DL, CKD, CRD, and AF, as well as age and sex, were simultaneously entered into a multivariable logistic regression model to investigate the impact of BMI on in-hospital mortality. Only odds ratios (ORs) and 95% confidence intervals (CIs) of each BMI category are shown. *AAD* acute aortic dissection, *AF* atrial fibrillation/flutter, *AHF* acute heart failure, *AMI* acute myocardial infarction, *BMI* body mass index, *CI* confidence interval, *CKD* chronic kidney disease, *CRD* chronic respiratory disease, *DM* diabetes mellitus, *DL* dyslipidemia, *HT* hypertension, *ICH* intracerebral hemorrhage, *IS* ischemic stroke, *OR* odds ratio, *SAH* subarachnoid hemorrhage.

We further performed multilevel mixed-effect logistic regression analysis after adjusting for age, sex, HT, DM, DL, CKD, CRD, and AF (model 2) to investigate the impact of BMI on in-hospital mortality and obtained similar results to those of model 1 (Fig. 4); the underweight group was consistently associated with a significantly higher risk of in-hospital mortality in six CVDs than the normal BMI group (AHF, OR 1.41, 95% CI 1.35–1.48, P < 0.001; AMI, OR 1.27, 95% CI 1.21–1.34, P < 0.001; AAD, OR 1.23, 95% CI 1.16–1.32, P < 0.001; IS, OR 1.45, 95% CI 1.41–1.50, P < 0.001; ICH, OR 1.18, 95% CI 1.13–1.22, P < 0.001; SAH, OR 1.17, 95% CI 1.10–1.26, P < 0.001). The overweight at risk group showed various ORs for in-hospital mortality: AHF, OR 0.93, 95% CI 0.89–0.97, P < 0.001; AMI, OR 1.01, 95% CI 0.97–1.06, P = 0.58; AAD, OR 1.10, 95% CI 1.04–1.17, P = 0.001; IS, OR 0.86, 95% CI 0.83–0.89, P < 0.001; ICH, OR 0.93, 95% CI 0.90–0.97, P = 0.001; SAH, OR 1.05, 95% CI 0.98–1.11, P < 0.15. In the obese I group, patients with AMI (OR 1.17, 95% CI 1.12–1.22, P < 0.001), AAD (OR 1.34, 95% CI 1.26–1.42, P < 0.001), ICH (OR 1.06, 95% CI 1.01–1.10, P < 0.001), and SAH (OR 1.27, 95% CI 1.20–1.34, P < 0.001) exhibited a significantly more positive relationship with in-hospital mortality than those in the normal BMI group. However, obese I patients with AHF and IS exhibited a significantly more negative relationship with in-hospital mortality than normal BMI patients (OR 0.91, 95% CI 0.87–0.96, P < 0.001 and OR 1.45, 95% CI 1.41–1.50, P < 0.001, respectively). In the four diseases for which the obese I group showed a significantly higher risk than the normal group, obese II patients had a higher risk for in-hospital mortality than normal BMI patients (AMI, OR 1.65, 95% CI 1.52–1.79, P < 0.001; AAD, OR 1.83, 95% CI 1.63–2.05, P < 0.001; ICH, OR 1.26, 95% CI 1.18–1.34, P < 0.001; SAH, OR 1.44, 95% CI 1.29–1.60, P < 0.001). Conversely, in AHF and IS, the obese II group did not show a significantly lower or higher OR for in-hospital mortality than the normal BMI group (AHF, OR 0.94, 95% CI 0.86–1.03, P = 0.16; IS, OR 1.03, 95% CI 0.95–1.11, P = 0.52). As the obese II group did not show a significantly lower risk of death, a clear obesity paradox was not observed for AHF and IS.

As in-hospital mortality was considered associated with age and age was negatively associated with a higher BMI category, we evaluated the impact of age in each BMI category in model 2 (Supplementary Fig. 2). Interestingly, for all CVDs, no age or BMI group showed a significantly lower OR than the reference 20 ≤ age < 65 years group with normal BMI. Age was significantly associated with a higher OR in every BMI category for all CVDs.

## Discussion

The present study analyzed hospitalized patients with six types of acute CVDs using a nationwide in-patient database and explored the BMI trends over time and the effect of BMI on outcomes of patients with CVDs. The main findings of this study are as follows: (i) age negatively correlated with higher BMI categories in six acute types of CVDs, (ii) the proportion of obese patients in the six types of acute CVDs gradually increased over time, and (iii) the underweight group demonstrated significant association with a higher risk of in-hospital mortality in six types of acute CVDs than the normal weight and overweight at risk groups; however, obesity showed a significant positive association with in-hospital mortality for AMI, AAD, ICH, and SAH but not for AHF and IS.

Generally, both obesity and underweight are associated with an increased risk of developing CVD and poor outcomes^5,21,22^. However, as the prevalence of patients with obesity and underweight and its severity are completely different for western countries^21,22^ and Asian countries^5^, data on Asian patients, stratified using the WHO Asian-BMI classification, are necessary to understand the current situation of BMI distribution in CVDs and the impact of underweight and obesity on mortality in Asian population. Until now, BMI trends in patients with CVD and the impact of BMI on death during hospitalization have remained poorly understood in Japan. Thus, this is the first report to provide information for six major acute types of CVDs using the JROAD-DPC dataset—one of the largest nationwide databases. The strength of this study is data reliability, due to the large sample size with systematically collected nationwide data using a uniform reporting format although we are not able to mention causality between BMI and mortality due to the nature of the study. Furthermore, as CVD is associated with aging and increase in aging populations is a global concern, it is important to establish clinical evidence for Japanese patients with CVD, the world’s most aged patients. Our results show that the mean age of patients with AHF was approximately 80 years, indicating that our data could be a good panorama reflecting an aging society.

Our data clearly show that age was inversely related with higher BMI categories in six acute types of CVDs. This trend was consistent with that observed in other large clinical studies conducted in Asian countries, such as Japan and China^18,23^. Considering that older individuals develop inflammageing that carries a high susceptibility to frailty—a leading contributor to functional decline in older adults^24^, this inverse relation is accurately reflected in real-world clinical settings. We observed a unique characteristic in the underweight group where the proportion of women were higher than that of men. This supports our idea that underweight patients show increased age-related physical frailty because frailty is more prevalent in women^25^. Conversely, patients with obesity in the six CVD groups were more likely to have lifestyle-related diseases such as HT, DM, and DL than those with normal weight and underweight, suggesting metabolic unhealthy conditions in obese patients. Besides BMI, assessment of fat mass may help to see the impact of obesity on mortality multidirectionally.

Analysis of time-dependent changes of BMI categories revealed similar 8-year-trends of AHF, AMI, and AAD and 5-year-trends of IS, ICH, and SAH; the proportion of obese patients gradually increased over time (Fig. 2). However, the proportion of the obese II category was < 7% in the six CVDs, a considerably lower percentage than that in data from western countries^3^. This low percentage of obese II patients was natural considering that the prevalence of obesity was only 3.8% in the general Japanese population^26^. Unexpectedly, the proportion of underweight patients did not significantly increase annually. This was due to the increased proportion of patients with obesity. These results highlight the importance of preventing obesity to inhibit the incidence of CVD. However, one report using a Japanese cohort showed that national health guidance intervention was not associated with clinically meaningful weight loss and cardiovascular risk factor reduction^27^. We, therefore, recommend determining specific designs of lifestyle interventions that are effective in improving obesity and lifestyle-related diseases.

An important result of this study is the impact of BMI on in-patient survival. After multivariable adjustment, underweight was strongly associated with an increased risk of in-hospital mortality in the six CVDs. This phenomenon was reproducible, as observed in another study conducted in China^23^. This may be because low BMI is associated with advanced age, and it reflects frailty—characterized by exaggerated decline in function and reserve of multiple physiological systems, resulting in a lower homeostatic tolerance of stressors and increased sensitivity and vulnerability to several adverse outcomes^28,29^. As the prevalence of the underweight group in the Asian populations is higher than that in the western populations^30^, we believe that data on underweight population are necessary to highlight that being underweight can adversely affect health. The overweight at risk population showed slightly better outcomes than normal weight patients with AHF, IS, and ICH but not those with AAD. To our knowledge, this is the first report demonstrating the risk burden of "overweight at risk” BMI with six CVDs on in-hospital mortality in Japanese patients. Although our results show that overweight at risk did not increase mortality risk in short-term outcomes, it does not imply that being overweight is beneficial; overweight is associated with an increased relative risk and population attributable risk for CVDs during long-term follow-up^31^. The fact the we used a normal BMI group as a reference for the in-hospital odds ratio needs to be borne in mind. The normal group consists of individuals with BMI from 18.5 kg/m^2^; they are thinner than average and are considered to have a higher in-hospital mortality rate than patients with 22–23 kg/m^2^ BMI. Restricted cubic splines analysis may help illustrate the dose–response association between BMI and in-hospital mortality. The obese I group exhibited worse outcomes in AMI, AAD, ICH, and SAH. This trend became more remarkable in obese II for these four diseases, indicating that obesity is associated with a higher mortality than normal BMI because it disturbs hemostasis via physiological mechanisms, such as increased inflammation and insulin resistance^1,32^. Interestingly, in AHF, the obese I group showed lower OR for in-hospital mortality; additionally, the obese II group did not show increased OR. This is the “obesity paradox” frequently observed in this short-term follow-up study in patients with heart failure. Although the underlying pathophysiological mechanisms remain unclear, various explanations have been proposed for the obesity paradox in patients with heart failure^33,34^. Altogether, this study provides evidence that the impact of underweight, overweight, and obesity on in-hospital mortality differs among acute types of CVDs. We believe that our data is helpful for general patient care in daily clinic. In the Japanese population, a representative of the Asian super-aged society, the burden of obese and underweight populations should be considered in patients with CVD.

### Limitations

This study has notable limitations. First, we analyzed only patients hospitalized in specific teaching hospitals contributing to the JROAD-DPC database. It only encompasses around 70% of the cardiovascular hospitals in Japan^16^, which may have led to a selection bias. Second, we did not distinguish metabolically healthy but obese phenotypes and metabolically abnormal obese phenotypes due to the lack of laboratory data in this DPC dataset. Recently, several studies have shown that the classification of obesity by BMI alone does not provide adequate insight into the potential risk of future adverse events as there is a metabolically healthy but obese phenotype^35,36^. That the metabolically healthy but obese phenotype alone did not significantly increase the subsequent CVD risk in Japanese patients should be considered when interpreting our results^37^. Third, there may be a possibility that we analyzed the information of one patient more than once since the same patient was assigned different identifiers when admitted to different hospitals. Fourth, we did not use standardized proportions because reports of Japanese Ministry of Health, Labour and Welfare are only available with the number of limited medical facilities and are not categorized by each diagnosis. Therefore, we did not calculate standardized proportions due to huge bias of its population denominator compared to our analyzed dataset. Finally, disease subtypes (e.g., heart failure with preserved or reduced ejection fraction, ST-elevation or non-ST-elevation myocardial infarction) could influence in-hospital mortality. However, we could not distinguish disease subtypes because of the limited DPC dataset. Further studies are warranted to bolster our data and identify the link between obese phenotypes and mortality in patients with CVDs.

## Conclusions

In this nationwide observational study, our results demonstrated the safety range of BMI for patients with CVDs with regard to in-hospital mortality. They may help promote medical policies focusing on BMI to prevent CVD. As the framework of the present study may be duplicated in future by other Asian countries with aging societies, we believe that our results have clinical significance and provide valuable information to fight against CVDs.

## Supplementary Information

Supplementary Information.

## Data Availability

The datasets generated and/or analysed during the current study are not publicly available due to the sensitive nature of the data collected for this study but are available from the corresponding author on reasonable request. All data supporting the findings of our study are available from Tomoya Yamashita (tomoya@med.kobe-u.ac.jp).

## Abbreviations

AAD: Acute aortic dissection
AF: Atrial fibrillation/flutter
AHF: Acute heart failure
AMI: Acute myocardial infarction
BMI: Body mass index
CKD: Chronic kidney disease
CRD: Chronic respiratory disease
CVDs: Cardiovascular diseases
DL: Dyslipidemia
DM: Diabetes mellitus
HT: Hypertension
ICD-10: International Classification of Disease and Related Health Problems 10th Revision
ICH: Intracerebral hemorrhage
IS: Ischemic stroke
JCS: Japanese Circulation Society
JROAD-DPC: Japanese Registry of All Cardiac and Vascular Diseases and the Diagnosis Procedure Combination
NYHA: New York Heart Association
SAH: Subarachnoid hemorrhage

## References

[1] Van Gaal LF, Mertens IL, De Block CE (2006). Mechanisms linking obesity with cardiovascular disease. Nature.

[2] Kivimäki M, Kuosma E, Ferrie JE, Luukkonen R, Nyberg ST, Alfredsson L (2017). Overweight, obesity, and risk of cardiometabolic multimorbidity: Pooled analysis of individual-level data for 120 813 adults from 16 cohort studies from the USA and Europe. Lancet Public Health.

[3] GBD. Obesity collaborators. Health effects of overweight and obesity in 195 countries over 25 years. *N. Engl. J. Med.* 2017 2015; 377:13–27. 10.1056/NEJMoa1614362 PMC5477817 28604169

[4] Poirier P, Giles TD, Bray GA, Hong Y, Stern JS, Pi-Sunyer FX (2006). Obesity and cardiovascular disease: pathophysiology, evaluation, and effect of weight loss: An update of the 1997 American Heart Association Scientific Statement on Obesity and Heart Disease from the Obesity Committee of the Council on Nutrition, Physical Activity, and Metabolism. Circulation.

[5] Zheng W, McLerran DF, Rolland B, Zhang X, Inoue M, Matsuo K (2011). Association between body-mass index and risk of death in more than 1 million Asians. N. Engl. J. Med..

[6] Blüher M (2019). Obesity: Global epidemiology and pathogenesis. Nat. Rev. Endocrinol..

[7] Lavie CJ, Laddu D, Arena R, Ortega FB, Alpert MA, Kushner RF (2018). Healthy weight and obesity prevention: JACC Health Promotion Series. J. Am. Coll. Cardiol..

[8] Lavie CJ, Milani RV, Ventura HO (2009). Obesity and cardiovascular disease: Risk factor, paradox, and impact of weight loss. J. Am. Coll. Cardiol..

[9] Yatsuya H, Li Y, Hilawe EH, Ota A, Wang C, Chiang C (2014). Global trend in overweight and obesity and its association with cardiovascular disease incidence. Circ. J..

[10] Chen Y, Copeland WK, Vedanthan R, Grant E, Lee JE, Gu D (2013). Association between body mass index and cardiovascular disease mortality in east Asians and South Asians: Pooled analysis of prospective data from the Asia Cohort Consortium. BMJ.

[11] Anker SD, Coats AJ (1999). Cardiac cachexia: A syndrome with impaired survival and immune and neuroendocrine activation. Chest.

[12] Balachandran A, de Beer J, James KS, van Wissen L, Janssen F (2020). Comparison of population aging in Europe and Asia using a time-consistent and comparative aging measure. J. Aging Health.

[13] Yasuda S, Miyamoto Y, Ogawa H (2018). Current status of cardiovascular medicine in the aging society of Japan. Circulation.

[14] Nakao K, Yasuda S, Nishimura K, Noguchi T, Nakai M, Miyamoto Y (2019). Prescription rates of guideline-directed medications are associated with in-hospital mortality among Japanese patients with acute myocardial infarction: A report From JROAD—DPC Study. J. Am. Heart Assoc..

[15] Yamashita Y, Morimoto T, Yoshikawa Y, Yaku H, Sumita Y, Nakai M (2020). Temporal trends in the practice pattern for venous thromboembolism in Japan: Insight From JROAD-DPC. J. Am. Heart. Assoc..

[16] Kanaoka K, Okayama S, Terasaki S, Nakano T, Ishii M, Nakai M (2020). Role of climatic factors in the incidence of takotsubo syndrome: A nationwide study from 2012 to 2016. ESC Heart Fail..

[17] Matoba T, Sakamoto K, Nakai M, Ichimura K, Mohri M, Tsujita Y (2021). Institutional characteristics and prognosis of acute myocardial infarction with cardiogenic shock in Japan—Analysis from the JROAD/JROAD-DPC Database. Circ. J..

[18] Itoh H, Kaneko H, Kiriyama H, Kamon T, Fujiu K, Morita K (2021). Reverse J-shaped relationship between body mass index and in-hospital mortality of patients hospitalized for heart failure in Japan. Heart Vessels.

[19] Ross JS, Normand SL, Wang Y, Ko DT, Chen J, Drye EE (2010). Hospital volume and 30-day mortality for three common medical conditions. N. Engl. J. Med..

[20] World Health Organization. *Regional Office for the Western P. The Asia-Pacific Perspective: Redefining Obesity and Its Treatment* (Health Communications, 2000).

[21] Bhaskaran, K., dos-Santos-Silva, I., Leon, D.A., Douglas, I.J., & Smeeth, L. Association of BMI with overall and cause-specific mortality: A population-based cohort study of 3^*.*^6 million adults in the UK. *Lancet Diabetes Endocrinol.* 2018; 6:944–953. 10.1016/S2213-8587(18)30288-2. 10.1016/S2213-8587(18)30288-2 PMC6249991 30389323

[22] Calle EE, Thun MJ, Petrelli JM, Rodriguez C, Heath CW (1999). Body-mass index and mortality in a prospective cohort of U.S. adults. N. Engl. J. Med..

[23] Deng F, Zhang Y, Zhao Q, Deng Y, Gao S, Zhang L (2020). BMI differences among in-hospital management and outcomes in patients with atrial fibrillation: Findings from the Care for cardiovascular Disease project in China. BMC Cardiovasc. Disord..

[24] Miyazawa I, Kadota A, Miura K, Okamoto M, Nakamura T, Ikai T (2018). Twelve-year trends of increasing overweight and obesity in patients with diabetes: The Shiga Diabetes Clinical Survey. Endocr. J..

[25] Ferrucci L, Fabbri E, Fabbri E (2018). Inflammageing: chronic inflammation in ageing, cardiovascular disease, and frailty. Nat. Rev. Cardiol..

[26] Clegg A, Young J, Iliffe S, Rikkert MO, Rockwood K (2013). Frailty in elderly people. Lancet.

[27] Fukuma S, Iizuka T, Ikenoue T, Tsugawa Y (2020). Association of the national health guidance intervention for obesity and cardiovascular risks with health outcomes among Japanese men. JAMA Intern. Med..

[28] Fried LP, Tangen CM, Walston J, Newman AB, Hirsch C, Gottdiener J (2001). Frailty in older adults: Evidence for a phenotype. J. Gerontol. A Biol. Sci. Med. Sci..

[29] Pandey A, Kitzman D, Reeves G (2019). Frailty is intertwined with heart failure: Mechanisms, prevalence, prognosis, assessment, and management’. JACC Heart Fail..

[30] NCD Risk Factor Collaboration (NCD-RisC). Worldwide trends in body-mass index, underweight, overweight, and obesity from 1975 to 2016: A pooled analysis of 2416 population-based measurement studies in 128·9 million children, adolescents, and adults. *Lancet.* 2017; 390:2627–2642. 10.1016/S0140-6736(17)32129-3. 10.1016/S0140-6736(17)32129-3 PMC5735219 29029897

[31] Wilson PWF, D'Agostino RB, Sullivan L, Parise H, Kannel WB (2002). Overweight and obesity as determinants of cardiovascular risk: The Framingham experience. Arch. Intern. Med..

[32] Gadde KM, Martin CK, Berthoud HR, Heymsfield SB (2018). Obesity: Pathophysiology and management. J. Am. Coll. Cardiol..

[33] Vest AR, Wu Y, Hachamovitch R, Young JB, Cho L (2015). The heart failure overweight/obesity survival paradox: The missing sex link. JACC Heart Fail..

[34] Carbone S, Canada JM, Billingsley HE, Siddiqui MS, Elagizi A, Lavie CJ (2019). Obesity paradox in cardiovascular disease: Where do we stand?. Vasc. Health Risk Manag..

[35] Ortega FB, Lee DC, Katzmarzyk PT, Ruiz JR, Sui X, Church TS (2013). The intriguing metabolically healthy but obese phenotype: Cardiovascular prognosis and role of fitness. Eur. Heart J..

[36] Smith GI, Mittendorfer B, Klein S (2019). Metabolically healthy obesity: Facts and fantasies. J. Clin. Invest..

[37] Itoh H, Kaneko H, Kiriyama H, Kamon T, Fujiu K, Morita K (2021). Metabolically healthy obesity and the risk of cardiovascular disease in the general population—Analysis of a nationwide epidemiological database. Circ. J..

