# Supplementary material for: Impact of body mass index on in-hospital mortality for six acute cardiovascular diseases in Japan
Source: Sci Rep. 2022 Nov 7;12:18934. doi: 10.1038/s41598-022-23354-y (PMC9640599; doi:10.1038/s41598-022-23354-y)
Supplement: Supplementary file 1 — Supplementary Information. [file 41598_2022_23354_MOESM1_ESM.pdf]

SUPPLEMENTARY MATERIAL

Impact of Body Mass Index on In-hospital Mortality for Six Acute Cardiovascular Diseases in Japan

Running Title: Body mass index and cardiovascular diseases

Naofumi Yoshida, MD, PhD[a†]; Masato Ogawa, PT, PhD[b,c†]; Michikazu Nakai, PhD[d]; Koshiro Kanaoka, MD, PhD[d]; Yoko Sumita[d]; Takuo Emoto, MD, PhD[a]; Yoshihiro Saito, MD[a]; Hiroyuki Yamamoto, MD, PhD[a]; Kazuhiro P. Izawa, PT, PhD[c]; Yoshitada Sakai, MD, PhD[b]; Yushi Hirota, MD, PhD[e]; Wataru Ogawa, MD, PhD[e]; Yoshitaka Iwanaga, MD, PhD[d]; Yoshihiro Miyamoto, MD, PhD[d]; Tomoya Yamashita, MD, PhD[a]; Ken-ichi Hirata, MD, PhD[a]

Affiliation:

[a]Division of Cardiovascular Medicine, Department of Internal Medicine, Kobe University Graduate School of Medicine, Kobe 6500017, Japan

[b]Division of Rehabilitation Medicine, Kobe University Hospital, Kobe 6500017, Japan

[c]Department of Public Health, Graduate School of Health Sciences, Kobe University, Kobe 6540142, Japan

[d]Department of Medical and Health Information Management, National Cerebral and Cardiovascular Center, Osaka 5648565, Japan.

[e]Division of Diabetes and Endocrinology, Department of Internal Medicine, Kobe University Graduate School of Medicine, Kobe 6500017, Japan

[†]These authors contributed equally

Corresponding author:

Tomoya Yamashita, MD, PhD

Division of Cardiovascular Medicine, Department of Internal Medicine,

Kobe University Graduate School of Medicine

7-5-1 Kusunoki-cho, Chuo-ku, Kobe 6500017, Japan

Phone: +81-78-382-5846

Fax: +81-78-382-5859

E-mail: tomoya@med.kobe-u.ac.jp

Supplementary Figure 1. Study population

Flowchart of the study for each cardiovascular disease.

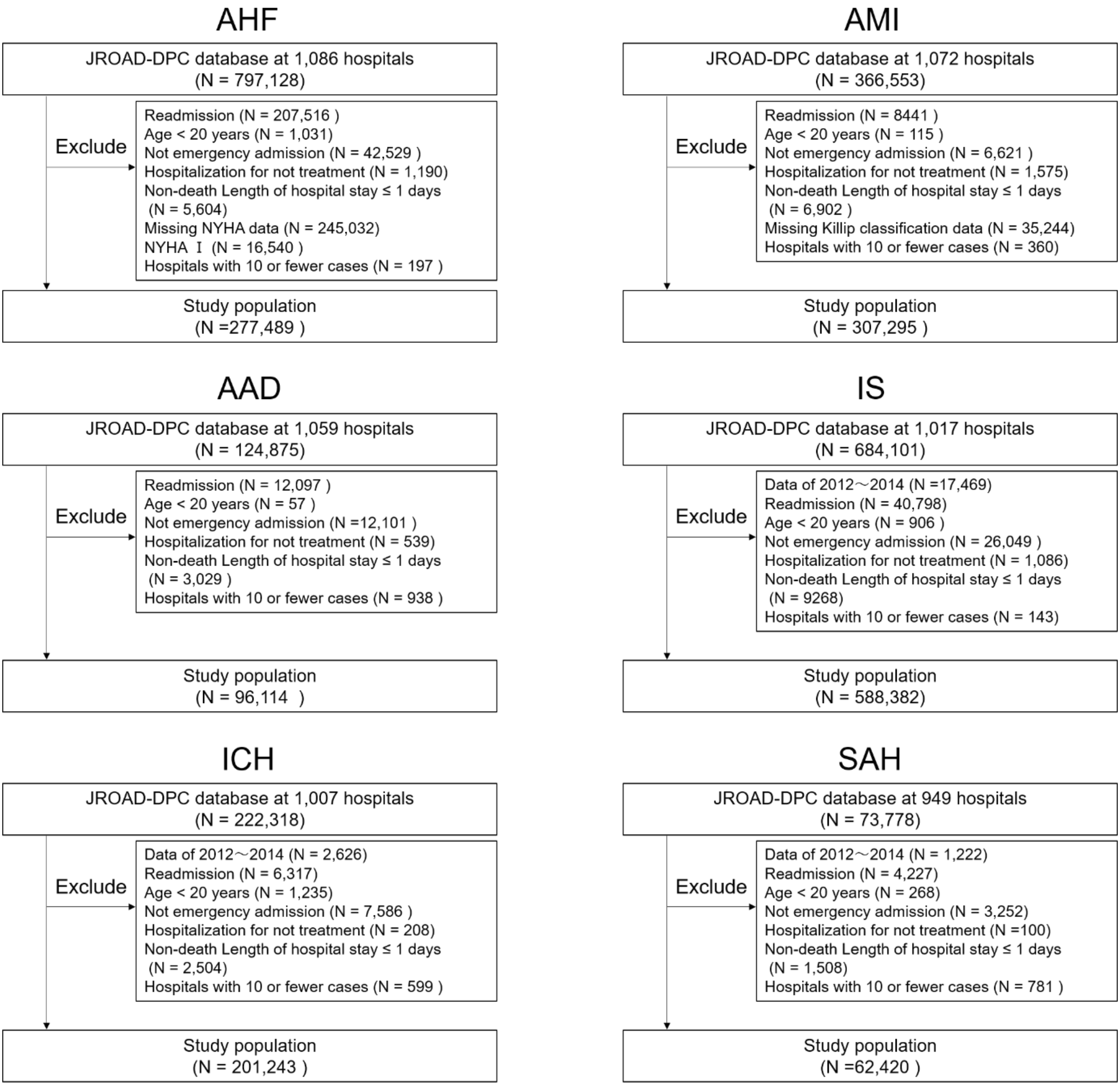

AAD, acute aortic dissection; AHF, acute heart failure; AMI, acute myocardial infarction; BMI, body mass index; ICH, intracerebral hemorrhage; IS, ischemic stroke; SAH, subarachnoid hemorrhage

Supplementary Figure 2. Interaction of P values for the indicated age subgroups

P value of interaction by subgroup analyses according to age in each BMI category.

AHF

	OR (95%CI)	P value
Underweight		
20 – 65 y.o.	1.91 (1.53-2.38)	<0.001
65 – 75 y.o.	2.83 (2.38-3.37)	<0.001
75 – y.o.	5.20 (4.55-5.93)	<0.001
Normal		
20 – 65 y.o.	1 (Ref)	-
65 – 75 y.o.	1.65 (1.43,1.91)	<0.001
75 – y.o.	3.66 (3.20,4.18)	<0.001
Overweight at risk		
20 – 65 y.o.	0.90 (0.71-1.13)	0.36
65 – 75 y.o.	1.48 (1.23-1.78)	<0.001
75 – y.o.	3.31 (2.89-3.79)	<0.001
Obese Ⅰ		
20 – 65 y.o.	0.87 (0.71-1.06)	0.16
65 – 75 y.o.	1.53 (1.30-1.81)	<0.001
75 – y.o.	3.11 (2.72-3.56)	<0.001
Obese Ⅱ		
20 – 65 y.o.	0.80 (0.63-1.01)	0.058
65 – 75 y.o.	1.45 (1.13-1.86)	0.004
75 – y.o.	2.96 (2.51-3.50)	<0.001

AMI

	OR (95%CI)	P value
Underweight		
20 – 65 y.o.	1.14 (0.92-1.41)	0.22
65 – 75 y.o.	2.24 (1.93-2.59)	<0.001
75 – y.o.	4.80 (4.35-5.29)	<0.001
Normal		
20 – 65 y.o.	1 (Ref)	-
65 – 75 y.o.	1.59 (1.45-1.74)	<0.001
75 – y.o.	3.63 (3.34-3.94)	<0.001
Overweight at risk		
20 – 65 y.o.	1.04 (0.93-1.17)	0.49
65 – 75 y.o.	1.69 (1.52-1.87)	<0.001
75 – y.o.	3.43 (3.14-3.75)	<0.001
Obese Ⅰ		
20 – 65 y.o.	1.27 (1.15-1.40)	<0.001
65 – 75 y.o.	1.84 (1.66-2.04)	<0.001
75 – y.o.	3.65 (3.33-4.01)	<0.001
Obese Ⅱ		
20 – 65 y.o.	1.59 (1.39-1.82)	<0.001
65 – 75 y.o.	2.63 (2.20-3.14)	<0.001
75 – y.o.	3.66 (3.08-4.35)	<0.001

AAD

	OR (95%CI)	P value
Underweight		
20 – 65 y.o.	0.99 (0.80-1.23)	0.92
65 – 75 y.o.	1.89 (1.62-2.21)	<0.001
75 – y.o.	3.62 (3.27-4.02)	<0.001
Normal		
20 – 65 y.o.	1 (Ref)	-
65 – 75 y.o.	1.47 (1.32-1.63)	<0.001
75 – y.o.	2.82 (2.57-3.11)	<0.001
Overweight at risk		
20 – 65 y.o.	1.27 (1.09-1.47)	0.002
65 – 75 y.o.	1.57 (1.39-1.78)	<0.001
75 – y.o.	2.93 (2.63-3.26)	<0.001
Obese Ⅰ		
20 – 65 y.o.	1.53 (1.37-1.70)	<0.001
65 – 75 y.o.	1.98 (1.76-2.23)	<0.001
75 – y.o.	3.11 (2.76-3.50)	<0.001
Obese Ⅱ		
20 – 65 y.o.	1.86 (1.61-2.15)	<0.001
65 – 75 y.o.	2.04 (1.59-2.62)	<0.001
75 – y.o.	3.83 (3.07-4.78)	<0.001

IS

	OR (95%CI)	P value
Underweight		
20 – 65 y.o.	1.83 (1.56-2.16)	<0.001
65 – 75 y.o.	3.20 (2.87-3.56)	<0.001
75 – y.o.	4.95 (4.55-5.38)	<0.001
Normal		
20 – 65 y.o.	1 (Ref)	-
65 – 75 y.o.	1.72 (1.57-1.88)	<0.001
75 – y.o.	3.38 (3.11-3.67)	<0.001
Overweight at risk		
20 – 65 y.o.	0.89 (0.77-1.02)	0.09
65 – 75 y.o.	1.43 (1.28-1.59)	<0.001
75 – y.o.	2.80 (2.57-3.05)	<0.001
Obese Ⅰ		
20 – 65 y.o.	1.01 (0.90-1.14)	<0.001
65 – 75 y.o.	1.51 (1.36-1.68)	<0.001
75 – y.o.	2.67 (2.45-2.92)	<0.001
Obese Ⅱ		
20 – 65 y.o.	1.09 (0.91-1.31)	0.35
65 – 75 y.o.	1.69 (1.42-2.02)	<0.001
75 – y.o.	2.82 (2.47-3.22)	<0.001

ICH

	OR (95%CI)	P value
Underweight		
20 – 65 y.o.	1.16 (1.06-1.28)	0.002
65 – 75 y.o.	1.54 (1.40-1.68)	<0.001
75 – y.o.	2.67 (2.51-2.85)	<0.001
Normal		
20 – 65 y.o.	1 (Ref)	-
65 – 75 y.o.	1.22 (1.15-1.30)	<0.001
75 – y.o.	2.28 (2.16-2.41)	<0.001
Overweight at risk		
20 – 65 y.o.	1.03 (0.94-1.12)	0.53
65 – 75 y.o.	1.16 (1.08-1.26)	<0.001
75 – y.o.	1.99 (1.86-2.13)	<0.001
Obese Ⅰ		
20 – 65 y.o.	1.15 (1.06-1.24)	0.001
65 – 75 y.o.	1.22 (1.12-1.32)	<0.001
75 – y.o.	2.21 (2.06-2.38)	<0.001
Obese Ⅱ		
20 – 65 y.o.	1.23 (1.12-1.36)	<0.001
65 – 75 y.o.	1.27 (1.06-1.51)	0.008
75 – y.o.	2.28 (1.96-2.66)	<0.001

SAH

	OR (95%CI)	P value
Underweight		
20 – 65 y.o.	1.10 (0.97-1.25)	0.14
65 – 75 y.o.	2.05 (1.81-2.32)	<0.001
75 – y.o.	4.96 (4.52-5.45)	<0.001
Normal		
20 – 65 y.o.	1 (Ref)	<0.001
65 – 75 y.o.	1.63 (1.50-1.77)	<0.001
75 – y.o.	4.15 (3.87-4.45)	<0.001
Overweight at risk		
20 – 65 y.o.	1.14 (1.04-1.25)	<0.001
65 – 75 y.o.	1.80 (1.61-2.01)	<0.001
75 – y.o.	3.79 (3.45-4.17)	<0.001
Obese Ⅰ		
20 – 65 y.o.	1.37 (1.26-1.49)	<0.001
65 – 75 y.o.	2.03 (1.82-2.27)	<0.001
75 – y.o.	4.44 (4.02-4.91)	<0.001
Obese Ⅱ		
20 – 65 y.o.	1.34 (1.17-1.53)	<0.001
65 – 75 y.o.	2.26 (1.77-2.90)	<0.001
75 – y.o.	4.92 (3.76-6.43)	<0.001

AAD, acute aortic dissection; AHF, acute heart failure; AMI, acute myocardial infarction; BMI, body mass index; CI, confidence interval; ICH, intracerebral hemorrhage; IS, ischemic stroke; OR, odds ratio; SAH, subarachnoid hemorrhage
